# Understanding medication oversupply and its predictors in the outpatient departments in Thailand

**DOI:** 10.1186/1472-6963-14-408

**Published:** 2014-09-19

**Authors:** Piyameth Dilokthornsakul, Nathorn Chaiyakunapruk, Piyarat Nimpitakpong, Napawan Jeanpeerapong, Katechan Jampachaisri, Todd A Lee

**Affiliations:** Department of Pharmacy Practice, Center of Pharmaceutical Outcomes Research, Faculty of Pharmaceutical Sciences, Naresuan University, Phitsanulok, Thailand; School of Pharmacy, Monash University Malaysia, Jalan Lagoon Selatan, Bandar Sunway, 47500 Selangor Malaysia; School of Population Health, University of Queensland, Brisbane, Australia; School of Pharmacy, University of Wisconsin-Madison, Madison, WI USA; Department of Pharmacy, Buddhachinaraj Hospital, Muang, Phitsanulok, Thailand; Department of Mathematics, Faculty of Sciences, Naresuan University, Phitsanulok, Thailand; Center for Pharmacoepidemiology and Pharmacoeconomic Research, College of Pharmacy, University of Illinois at Chicago, Chicago, IL USA

**Keywords:** Medication oversupply, Prevalence, Financial loss, Factors association, Thailand

## Abstract

**Background:**

Medication oversupply is an important problem in the healthcare systems. It causes unnecessary avoidable healthcare costs. Although some studies have determined the magnitude and financial loss due to medication oversupply in western countries, they may not be applicable to Asia-pacific countries. This study aims to determine the prevalence, financial loss, and patterns of medication oversupply and the factors associated with such oversupply in Thailand.

**Methods:**

A retrospective database analysis was used from 3 public hospitals. Patients visiting the outpatient department of the hospitals in 2010 and receiving at least 2 prescriptions within 6 months were included. The modified medication possession ratio (MPRm) was used to determine the medication supply. Patients having MPRm > 1.20 were defined as receiving a medication oversupply. The measures were prevalence of medication oversupply, the number of oversupplied medications, and financial loss (2012 dollars) due to medication oversupply. Hierarchical logistic regression was used to determine the factors associated with the prevalence of medication oversupply.

**Results:**

A total of 99,743 patients were included. Patients were on average 49.7 ± 21.2 years of age, and 42.8% were male. Most of them were adult (53.7%). Among those patients, 60.2% of the patients were under universal coverage schemes. Around 13.4% of all the patients received a medication oversupply, and the patients in regional hospitals had a higher prevalence of medication oversupply than patients in district hospitals (13.8% VS 8.2%). The patients under civil servant medical benefit schemes (CSMBS) (13.6%) had the most prevalence of medication oversupply. The total financial loss was $189,024 per year. The average financial loss was $1.9 ± 19.0 per patient/year. Patients under CSMBS experienced the highest average financial loss (2.6 ± 23.2 $/patient/year). Age, gender, health insurance schemes, and the number of medications that the patients received were the factors associated with medication oversupply.

**Conclusions:**

Medication oversupply is an important problem for the health system. Patients receiving care from regional hospitals had a higher likelihood of medication oversupply. Policymakers may consider developing policies for preventing medication oversupply. The policy should be implemented in regional hospitals and especially in children or patients with poly-pharmacy.

## Background

Many countries are facing substantial increases in drug expenditure [1]. This is likely attributable to multiple factors, including the advance of technology that has brought with it higher costs, the aging of the population, and changes in prescribing practices [2–6]. However, one of the important understudied factors is medication oversupply [7].

Medication oversupply occurs when patients receive an amount of medication that exceeds what they need [8]. It is an important problem in the healthcare systems in several countries. The prevalence of medication oversupply was 12%-22% [9, 10] in Sweden and 30%-47% in the USA [11–14]. In Thailand, the prevalence of medication oversupply has been estimated to range from 5.9% - 47.2% [7, 15].

Medication oversupply causes unnecessary avoidable healthcare costs. A study conducted in the USA indicated that patients receiving a medication oversupply spent more on medication than patients with a proper supply at around 14% [11] for only 12 medication class. In Sweden, the extra cost due to medication oversupply was approximately 4.3% of the total medication cost [16]. Furthermore, medication oversupply may increase the risk of adverse clinical consequences. Several studies have revealed that patients with a medication oversupply had higher risks of hospitalization than those with a proper supply [7, 9, 10, 17]. Further, a study conducted with patients with type 2 diabetics reported that patients receiving a medication oversupply were 5% less likely to achieve recommended levels of their hemoglobin A1C than patients with a proper supply [18].

Since the majority of previous studies were conducted in developed countries, the findings might not be applicable to developing countries. Healthcare systems in developing countries might be different from those of developed countries, including lack of controls in the supply system for medications. For example, in Thailand there is no utilization management procedure in place to discourage or prevent early or excessive refilling of medications. Day supply limits, which are common in developed countries, prevent excessive medication refills by not allowing excessive medication refills for patients and are not common in Thailand. Thus, the findings on medication oversupply in developed countries might not be applicable to developing countries, including Thailand where oversupply may be an even bigger problem for the healthcare system.

Although some prior studies have been conducted to determine the prevalence and financial burden due to medication oversupply in Thailand, they were conducted in a single hospital and focused on a limited number of medications [7, 15]. One of them was conducted at a university teaching hospital, while another was conducted at a regional hospital that focused only on the 5 medications that were responsible for the highest expenditures. In Thailand, there are 4 major types of hospitals: 1) university hospitals, which usually serve patients with very severe and complex diseases; 2) regional hospitals, which commonly serve patients with a wide-range of complex and severe diseases; 3) general hospitals, which also serve patients with any diseases of lower complexity and severity; and 4) district hospitals, which serve patients with non-complicated diseases. In 2010, the government hospitals in Thailand consisted of 93 regional/general hospitals covering all provinces in Thailand, 731 district hospitals, and over 9,700 Tumbon Health Promoting Hospitals (THPHs) [19] or sub-district health centers along with a network of urban and rural community primary healthcare centers and village health volunteer systems [20, 21]. Because of the differences in service among the types of hospitals, previous studies might not be generalizable to other types of hospitals. Moreover, previous studies did not identify the factors affecting medication oversupply, which could lead to targets for interventions. Thus, this study aimed to determine the patterns of medication oversupply, its financial burden, and its associated factors in Thailand.

## Methods

### Data sources

A retrospective database analysis was conducted from the healthcare payer perspective. Electronic databases obtained from 3 hospitals were used. In conducting this study, the authors had access to data from three hospitals in the northern part of Thailand. Two hospitals were regional hospitals, located in the northern part and northeastern part of Thailand. Another hospital was a district hospital located in the northern part of Thailand. In order to increase the generalizability of the findings, both regional and district hospitals, which serve a different case-mix of patients, were included. The electronic databases included demographic databases, outpatient diagnostic databases, and pharmacy databases. They contained information on age, sex, health insurance, date of birth, International Statistical Classification of Disease, 10^th^ Revision [ICD-10], visit date, drug name, drug code, drug regimen, and the amount of medication per prescription. This study was approved by the Naresuan University Human Ethics Committee and the ethics committee of each hospital which were Buddhachinaraj hospital, Sunpasitthiprasong hospital, and Nakhon Thai Crown Prince hospital.

### Patient and study period

The authors identified all of the patients visiting the outpatient departments (OPD) of the three hospitals in 2010. The inclusion criteria were the following: 1) patients visiting the OPD of the hospitals from Jan 1^st^ – Dec 31^st^, 2010; 2) patients receiving at least 2 prescriptions within 6 months for the same specific generic name of medications; 3) patients receiving any medication for chronic conditions. Patients were followed up from the first date of receiving the medications until the last of the medication dispensation or Jun 30^th^, 2011.

### Medication supply measurements

Medication supply was measured using the modified medication possession ratio (MPRm) for each medication that the patients received. The medications that patients received only one time were excluded from the analysis. The MPRm was calculated by dividing the total days’ supply of a specific generic name of medication for a patient by the number of days from first to last dispensation, plus the number of days’ supply in last dispensation. We defined our operational definition of medication oversupply (MPRm > 1.20) based on two reasons. First, we selected 120% to represent a potentially clinically significant oversupply of medications. Several studies indicated that the cut-off point of non-adherence should be 80% of perfect adherence (20% difference from perfect adherence), which affects the morbidity and mortality in several diseases [22–24]. We felt that a 20% difference from perfect adherence (100%) may also be meaningful in terms of medication oversupply. While there are no studies that have evaluated the clinical significance of this value, we felt that the absolute difference of 20% was a reasonable estimate for identifying someone with an oversupply of medication. Second, we considered the issue of medication supply management. It is reasonable that patients are allowed some additional medications to allow for medication loss or delayed refill. In the US insurance companies often allow for a 7-day overlap of a refill for a medication with a 30-day supply. The 7-day overlap allowance for a medication with a 30-day supply is about a 23% overlap. Based on these two reasons, we considered that the MPR > 1.20 (>20% overlap) was the cut-off value for determining medication oversupply. Patients were identified as having an appropriate supply when they had an MPRm between 0.80 -1.20 in all of the medications that the patients received. Since patients usually received more than one medication, we classified patients as having medication oversupply if at least one of the MPRms was >1.20, the cut-off which has been used in previous literatures [8, 11, 14, 16].

The MPRm was slightly modified from the medication possession ratio (MPR) by changing the denominator of the formula from a pre-specified time period (such as 1-year follow-up period) to the number of days from first to last dispensation, plus the number of days’ supply in the last dispensation [15, 25, 26].


**Figure 1:**
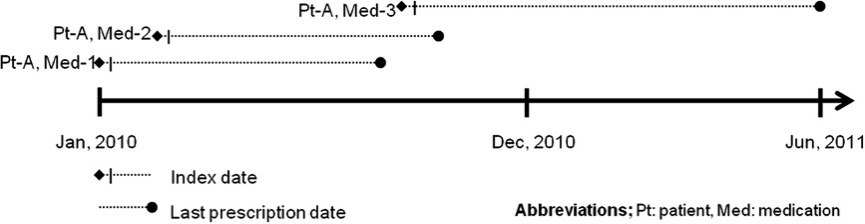
**Index date, patient tracing, and study period. Legend:** This figure illustrates how the patients in this study were followed up. For example, patient A received in total 3 medications (Med-1, Med-2, and Med-3). We followed patient A from Jan 2010 to Jun 2011 but we tracked data for medication 1 of patient A until the last dispensation date. We also tracked the data for each medication that patient A received. We calculated the modified medication possession ratio (MPRm) for each medication that patient A received based on the first and last date of dispensation. Thus, patient A had 3 MPRms. We classified patient A as oversupplied if any MPRm of patient A >1.20.

All of the patients were traced to determine the medication supply that each patient received. The index date of each medication was determined according to the first dispensation date of each specific medication. Patients that met the inclusion criteria were traced from the index date to the last dispensation date of each specific medication (Figure 1). For instance, patient A received 3 medications. The 1^st^ medication was dispensed from January 1^st^ to October 31^st^, 2010. The 2^nd^ medication was dispensed from February 1^st^ to November 30^th^, 2010. The last one was dispensed from November 15^th^, 2010 to June 30^th^, 2011. The patient was traced from January 1^st^, 2010 to June 30^th^, 2011 and the medication supplies of the patient were calculated for each specific generic name of medication.

The MPRm for each specific generic name of medication, except for transdermal medications and eye, ear, and nasal drops, was calculated in this study. We excluded transdermal medications, eye, ear, and nasal drop out of our study because they have no exact dose of administration and the MPRm could not be calculated. Medications prescribed for use as needed and immediate use were also excluded because they could not be used to calculate the MPRm.

### Outcome measures of interest

The primary outcome measure of interest was the prevalence of patients having a medication oversupply. Secondary outcome measures were the number of oversupplied medications, and financial loss due to medication oversupply. The prevalence of patients experiencing a medication oversupply was defined as the proportion of patients that had an MPRm > 1.20 over the total number of patients. Patients that had an MPRm 0.8-1.2 were identified as having an appropriate supply and patients that had an MPRm < 0.8 were identified as having an undersupply. The number of oversupplied pills was presented as the total number of oversupplied pills and the average number of oversupplied pills per patient. Financial loss due to medication oversupply was determined by the number of oversupplied pills multiplied by the reference costs of each medication purchased during the year 2012 [27]. Financial loss was converted to US$ using an exchange rate of 29.42 baht/US$ [28]. The cost was annualized to 1 year cost.

### Data analysis

Descriptive statistics were used to describe the baseline characteristics and outcome measures of interest. Sensitivity analysis was also performed by varying the definition of medication oversupply from MPRm 1.20 to 1.10. Subgroup analysis was performed by type of hospitals (regional vs. district), and health insurance (universal health coverage schemes (UCS), social security schemes (SSS), civil servant medical benefit schemes (CSMBS), and other insurances. We used the type of hospital for the subgroup analysis because different hospitals have different outpatient medication management systems. It may affect outcomes in this study. In our study, we used 2 types of hospital as regional and district hospitals. We also used health insurance for the subgroup analysis. In Thailand, there are 3 main health insurances, including the UCS, SSS, and CSMBS. Conceptually, the UCS and SSS employ capitation payment, while the CSMBS is fee-for-service payment. That might affect the patterns of the prescriptions prescribed and oversupply of medication. Chi-square statistic was used to examine the difference in the number of oversupplied patients among subgroups. The Mann–Whitney U-test or the Kruskal-Wallis test was used to examine the difference in financial loss among subgroups.

To determine the factors associated with medication oversupply, we used hierarchical logistic regression because the data structure we used fit the model. The individual-level data (lower-level) nested in the hospital level variable (upper-level). The dependent variable was having a medication oversupply for each patient. Patients having a medication oversupply were compared to the appropriately-supplied patients. The undersupplied-patients were not considered in the factor association analysis. The primary reason was that we were only interested in investigating the factors determining patients having an oversupply. Having an appropriate supply as a comparator would help us understand the differences between these two groups. In addition, we could treat oversupply as a dependent dichotomous variable. We used type of hospital as a random-effects variable and used age group (children/adolescent, adult, and elderly), gender, health insurance, Charlson’s co-morbidity index (an algorithm commonly used for assessing the comorbidity burden of patients) [29], and number of medications patients received (<5 vs ≥5 medications) as fixed-effects variables. All of the fixed-effects variables were included in the model as these were identified as important factors in our literature review. A generalized chi-square was used to assess the goodness of fit of the model (A generalized chi-square per degree of freedom <2.0 indicates no lack of fit).

## Results

**Figure 2:**
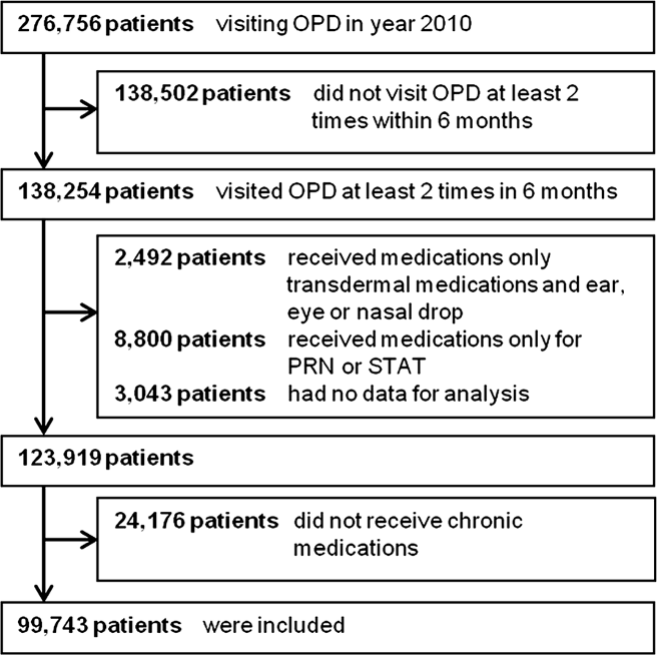
**The flow of patient selection.**

We identified 276,756 patients visiting an OPD in 2010. Among those, 99,743 patients met the inclusion criteria (Figure 2). Patients were on average 49.721.2 years of age with 42.8% being male. Most of them were adults (53.7%). Among those patients, 60.2% of the patients were under a UCS. The detailed baseline characteristics are shown in Table 1. The pattern of medication supply is shown in Table 2. The percentage of patients that had a medication oversupply was 13.4%, while 47.7% of the patients had an appropriate supply. On average, the number of days’ supply was 354.7 ± 247.3 days for oversupplied patients and 371.1 ± 185.1 for appropriate supplied patients. The average number of medication items received was 6.2 ± 4.4 and 3.9 ± 2.7 for oversupplied and appropriately-supplied patients, respectively.

**Table 1:** **Baseline characteristics**

Patient characteristics	Number of patients (%)
	(N = 99,743)
***Type of hospital***	
Regional hospital	91,614 (91.9)
District hospital	8,129 (8.2)
***Age*** **(Mean ± SD)**	49.7 ± 21.2
Children/adolescent (<18 years old)	10,799 (10.8)
Adult (18–59 years old)	53,564 (53.7)
Elderly (≥60 years old)	35,380 (35.5)
***Gender***	
Male	42,651 (42.8)
Female	56,860 (57.0)
Missing data	232 (0.2)
***Health insurance***	60,026 (60.2)
Universal health coverage schemes	7,760 (7.8)
Social security schemes	29,203 (29.3)
Civil servant medical benefit schemes	2,522 (2.5)
Others	232 (0.2)
Missing data	
***Charlson’s co-morbidity index***	1.1 ± 1.7
Mean **±** SD	51,073 (51.2)
0	36,107 (36.2)
1-2	12,312 (12.3)
>3	251 (0.3)
Missing data	

*Abbreviations*: *SD* standard deviation.

**Table 2:** **The patterns of medication supply**

Variables	Undersupply	Appropriate	Oversupply
	(<0.8)	supply (0.8-1.2)	(>1.2)
Number of patients (%)	38,881 (39.0)	47,527 (47.7)	13,324 (13.4)
Average day supply (days)	260.7 ± 185.1	371.1 ± 185.1	354.7 ± 247.3
Average follow-up time (days)	334.4 ± 169.3	365.9 ± 180.1	331.0 ± 18.6
Average MPRm	0.6 ± 0.3	1.0 ± 0.7	1.2 ± 0.6
Average number of medication receipts	5.2 ± 3.4	3.9 ± 2.7	6.2 ± 4.4
Average oversupplied items per patient	N/A	N/A	0.3 ± 0.5

*Abbreviations*: *MPRm* modified medication possession ratio, *N/A* not applicable.

### Magnitude of medication oversupply

There were 13.4% of patients that had a medication oversupply (13,330/99,743 patients). The total oversupplied pills/units were 785,223 pills/units or 7.9 ± 68.6 pills/units per patient per year. The prevalence of medication oversupply was 22.7% when the definition of medication oversupply was changed to MPRm >1.10 (Table 3).

**Table 3:** **Magnitudes and financial loss due to medication oversupply**

Variables	Prevalence of	Total oversupplied	Average oversupplied	Total financial loss	Average financial loss
	oversupplied	pills or units	pills or units/patients	per year (US$)	(US$/patient/year)
	patients (%)				
MPRm >1.20	13.4	785,223	7.9 ± 68.6	189,024	1.89 ± 19.04
MPRm >1.10	22.7	1,584,955	15.9 ± 91.8	307,552	5.24 ± 31.61
**By type of hospital (MPRm > 1.20)**					
Regional hospital	13.8	771,727	8.4 ± 71.1	93,030*	2.03 ± 19.82
District hospital	8.2	13,496	1.7 ± 28.4	2,963	0.36 ± 4.14
**By health insurance (MPRm > 1.20)**					
UCS	13.4	470,620	7.8 ± 66.7	101,280	1.7 ± 18.1
SSS	11.6	34,976	4.5 ± 37.7	8,669	1.1 ± 9.0
CSMBS	13.6	249,456	8.5 ± 60.5	76,030	2.6 ± 23.2
Others	13.3	28,719	11.4 ± 181.6	2,810	1.1 ± 8.1
Missing	23.7	1,452	6.7 ± 23.1	234	1.0 ± 5.5

*Abbreviations: CSMBS* civil servant medical benefit schemes, *MPRm* modified medication possession ratio, *SSS* social security schemes, *UCS* universal coverage schemes.

*Financial loss per hospital.

The prevalence of medication oversupply in the regional hospitals was higher than that in the district hospitals (13.8% VS 8.2%; *p* < 0.001). The problem of medication oversupply was slightly different among different health insurance schemes. Among the patients under the UCS, 8,061 of 60,026 (13.4%) patients had a medication oversupply, while the patients under the SSS and CSMBS were at 11.6% and 13.6%, respectively (*p* < 0.001) (Table 3).

### Financial loss due to medication oversupply

The total financial loss due to medication oversupply was $189,024 per year or $1.9 ± 19.0 per patient/year. The overall medication expenditure of the entire cohort was $16,533,401. The total financial loss due to medication oversupply accounted for 1.1% of total outpatient drug expenditure. When the definition of medication oversupply was changed to MPRm > 1.10, the financial loss increased to $307,552 (1.9% of total expenditure). The average financial loss was 1.9 ± 9.0 and 5.2 ± 31.6 $/patient/year for MPRm >1.20, and >1.10, respectively (Table 3).

Financial loss due to medication oversupply at the regional hospitals was higher than that of the district hospitals. The total financial loss at the regional hospitals was 93,030 $/hospital, while the total financial loss at the district hospitals was 2,963 $/hospital. The average financial loss was 2.0 ± 19.8 $/patient/year in the regional hospitals, while the average financial loss was 0.4 ± 4.1 $/patient/year in the district hospitals (*p* < 0.001)(Table 3). The average financial loss for patients under the CSMBS (2.6 ± 23.2 $/patient/year) was higher than that of patients under the UCS (1.7 ± 18.1 $/patient/year) and SSS (1.1 ± 9.0 $/patient/year) (*p = 0.004)*.

### Factor associated with medication oversupply

The generalized chi-square per degree of freedom was 1.01, which indicated no lack of fit. Based on our multivariate hierarchical logistic regression model, children and adolescent patients had a higher risk of being oversupplied with medications than adult patients (adjusted odds ratio (AOR) 3.303; 95% confidence interval (CI), 3.095 – 3.525)), while elderly patients tended to have a higher risk compared to adult patients but this was not statistically significant (AOR 1.039 (95% CI; 0.992- 1.088)). Female patients were at higher risk of being oversupplied with medications than male patients (AOR; 1.177 (95% CI; 1.131-1.226). Patients under the SSS were at higher risk compared to patients under the UCS (AOR 1.200 (95%CI; 1.106 – 1.302), but the patients under the CSMBS had a similar risk of medication oversupply compared to patients under the UCS (AOR 1.030 (0.982-1.079)). The patients that received medications 5 items or more (also called poly-pharmacy) were at higher risk of being oversupplied with medications than patients that received fewer than 5 medications (AOR 2.625 (95%CI; 2.507-2.748)). Patients visiting regional hospitals tended to receive more oversupplied medication than patients visiting district hospitals, but this was not statistically significant (AOR 1.235 (95%CI; 0.056 – 27.027)) (Table 4).

**Table 4:** **Factors associated with medication oversupply (MPR >1.20)**

Factors	Adjusted odds ratio
	(95% CI)
***Age group***	
Adult (18–59 years old)	*Reference*
Children/Adolescent (<18 years old)	3.303 (3.095 - 3.525)
Elderly (≥60 years old)	1.039 (0.992 – 1.088)
***Gender***	
Male	*Reference*
Female	1.177 (1.131 – 1.226)
***Health insurance***	
UCS	*Reference*
SSS	1.200 (1.106 – 1.302)
CSMBS	1.030 (0.982 – 1.079)
Others	1.075 (0.946 – 1.221)
***Charlson’s co-morbidity index***	1.067 (1.055 – 1.079)
***Number of medication patients received***	
<5 medications	*Reference*
≥5 medications	2.625 (2.507 – 2.748)
***Type of hospital***	
District hospital	*Reference*
Regional hospital	1.235 (0.056 – 27.027)

*Abbreviations*: *CSMBS* civil servant medical benefit schemes, *SSS* social security schemes, *UCS* universal coverage schemes.

## Discussion

This study demonstrated that medication oversupply is an important problem in the healthcare system in Thailand. Around one-seventh of patients had an excess supply of medications based on the days’ supply they were dispensed and their government experienced unnecessary financial loss due to this medication oversupply.

Comparing the prevalence of patients having medication oversupply in our study with that in a previous Thai study conducted in a teaching hospital, the prevalence found in our study was around 2.3 times higher. There were likely two possible reasons for this. First, the definition of medication oversupply in our study differed from that in the previous study. The definition of medication oversupply in this study was MPRm > 1.20, while in the other study it was more than 30 days left. Second, the drug utilization patterns in regional and district hospitals might be different from those in teaching hospitals. A teaching hospital usually has a large number of medical specialists and specialty clinics that may cause differences in drug utilization patterns compared to other types of hospitals. In addition, the prevalence of patients having a medication oversupply in the teaching hospital is relatively low because patients visiting a teaching hospital might not adhere to care at the hospital—they may seek care at other hospitals close to their homes.

The total financial loss due to medication oversupply in this study was not substantial. The financial loss was 1.1% of total OP drug expenditure. This finding was different from that of Kaojarern’s study [15], which reported that the financial loss accounted for 2.6% of total oral OP drug expenditure. This was likely due to the difference in definition of medication oversupply. Although the total financial loss was not tremendous, it would be better for the government to prevent unnecessary financial loss due to medication oversupply and spend the saved money to improve the healthcare system. Moreover, it is important to note that our study determined only direct financial loss. The indirect financial loss due to medication oversupply might also affect the government’s budget. Again, the unnecessary financial loss due to medication oversupply could be preventable.

The subgroup analysis indicated that regional hospitals had a higher prevalence of patients having a medication oversupply and also greater financial loss. This difference was likely due to the large number of patients visiting the hospitals, which leads to a greater workload on the part of healthcare workers. Because of this high workload, healthcare providers might not pay attention to reviewing the amounts of medications dispensed. Moreover, patients with more complex diseases and the more sophisticated health service systems in the regional hospitals might affect the quality of care in those hospitals.

Another subgroup analysis demonstrated that there was not much difference in the prevalence of patients having a medication oversupply across different types of health insurance. However, the average financial loss due to medication oversupply in patients under the CSMBS was about 2 times higher than patients under the UCS and SSS. This was likely due to the mechanism of reimbursement. The hospitals can be fully reimbursed by direct billing to the comptroller general's department, which is a government organization paying for patients under the CSMBS. Since providers have no concerns about the financial burden to patients, they may prescribe higher-cost medications to patients under the CSMBS. Moreover, patients have no concern about the amount of medications prescribed because there is no out-of-pocket cost for them.

After adjustment for other variables in the same regression, there were 2 factors which were strongly associated with having a medication oversupply: age and number of medications. Children had a 3.3 higher risk of having a medication oversupply than the adults non-elderly patients. The prevalence of children visiting hospitals was likely to be greater than adult patients. The patients visiting the hospital more often were likely to receive the same medications that they received previously than patients visiting the hospital less often. That might increase the chance of having a medication oversupply. The second factor associated with medication oversupply was using more than 5 medications. Patients receiving at least 5 medications had a 2.6 higher risk of having a medication oversupply than patients receiving fewer than 5 medications. It is possible that the patients receiving more medications had more chronic conditions and potentially more severe conditions; they therefore might need to visit the hospital more often than patients with less severe diseases. Again, the patients visiting the hospital more often are likely to receive the same medications that they received previously than patients visiting the hospital less often, which will increase the chance of experiencing a medication oversupply.

There are other factors which might cause a medication oversupply than the factors we found in this study. The poor medication management system might be a major cause of medication oversupply. The medication management system, such as outpatient medication reconciliation, might not be appropriately implemented. Thus, there might be no information about the amount of medication that the patients received. Lack of an appropriate communication system among healthcare providers may be another major cause—it increases the chance of receiving the same medications from different clinics, which can result in medication oversupply.

In this study, we used the MPRm > 1.20 as our operational definition. This definition allows 20% excessive amount of medication supply for each patient. It helps policy makers to better understand the magnitude of the problem and its financial loss when they allow some additional medication for patient to cover their medication loss or delayed refill. Even though, patients are allowed to receive 20% excessive medication, there are still about 14% of patients had medication oversupply. The definition emphasized inappropriately over-excessive amount of the medication patients received and indicated inappropriate financial loss due to medication oversupply. Policy makers might use our findings to consider how much the budget might be saved when considering only over-excessive medication oversupply.

Several strategies or policies could potentially be implemented for preventing medication oversupply. An effective outpatient medication reconciliation and medication refill system should be considered because they are not costly strategies but are likely to be effective. A previous study [30] conducted to determine potential strategies to prevent unnecessary loss due to medication oversupply in Thailand also suggested that an effective outpatient medication reconciliation and medication refill system should be considered. Moreover, those strategies may be useful for other purposes such as preventing inappropriate medication use or continuity of medication use. Because of a high prevalence of medication oversupply and also high financial burdens at regional hospitals, the above-mentioned strategies should be implemented at regional hospitals. Specifically, children or patients with poly-pharmacy should be targeted since they are more likely to have medication oversupply than others.

Another strategy suggested for preventing medication oversupply is an effective computer system, one that is set up to detect medication oversupply. However, this system is likely to be more expensive than the outpatient medication reconciliation or medication refill system. The computer system should be considered later if outpatient medication reconciliation or medication refill systems are not effective.

Some limitations of this study should be recognized. First, these findings were based on the electronic databases of 3 hospitals and might not be representative of all hospitals in Thailand. The generalizabilty of these current findings to other hospitals is limited and interpretation should be done with cautions. Second, these findings relied on electronic databases and a misclassification could have occurred. However, these hospitals have used the data for claiming purposes and we believe that the data used in this study were credible. Moreover, the databases from the district hospital were assessed for the quality of data, resulting in both high specificity and sensitivity. Third, the definition of patients receiving medication oversupply that we used was that patients had a medication oversupply if the MPRm for any medication was >1.20. The result would likely have been different if we used a different definition of patients receiving a medication oversupply. However, we decided to use this definition because we thought that any medication that patients were oversupplied with reflected the fact that there were some problems in the system, leading to the lack of ability to detect the excessive amount of medication that the patients received. Last, because of data limitation we could not fully understand the reasons behind the high burden in regional hospitals. This warrants future research investigation.

## Conclusion

This study emphasized that medication oversupply is an important problem in Thailand. Policy-makers should consider the development of policies to decrease the problem. Further policies might focus on regional hospitals and patients under the CSMBS.

## Abbreviations

AOR: Adjusted odds ratio
CSMBS: Civil servant medical benefit schemes
MPRm: Modified medication possession ratio
OPD: Outpatient department
SSS: Social security schemes
UCS: Universal coverage schemes.
